# A Scalable Droplet Microfluidic Platform That Overcomes Large‐Cell Constraints for Long‐Term Plant Cell Culture and Embryogenesis Analysis

**DOI:** 10.1002/advs.77936

**Published:** 2026-09-27

**Authors:** Arman Mirmiran, Fangchi Shao, Gregory Datto, Tathansh Joshi, Elizabeth Chatt, Yashar Bashirzadeh, Dan Stessman, Lai Wei, Kuangwen Hsieh, Hena Guo, Tza‐Huei Wang

**Affiliations:** ^1^ Department of Mechanical Engineering Johns Hopkins University Baltimore USA; ^2^ Department of Biomedical Engineering Johns Hopkins University Baltimore USA; ^3^ Corteva Agriscience Johnston Iowa USA

**Keywords:** doubled haploid breeding, droplet microfluidics, high‐throughput culture, large plant cells, maize microspores, microspore‐derived embryogenesis, viability assessment

## Abstract

Microspore‐derived embryogenesis (MDE) is essential for doubled haploid (DH) plant breeding, but conventional bulk culture systems are limited by poor scalability and difficulty for automation. Adapting droplet microfluidics for plant cell culture is promising but challenging due to the large size and fragility of plant cells involved in embryogenesis, such as maize microspores (60–85 µm) and their expanding embryo‐like structures (200–500 µm). Here, we present a droplet microfluidic platform engineered for large plant cells, integrating a tubing‐based loading strategy that minimizes sedimentation losses, robust generation of stable 20 and 200 nL droplets optimized for large‐cell encapsulation, and scalable 3D reservoir incubation for long‐term culture. Optimized flow conditions and surface treatments enabled cell encapsulation with high fidelity, sustaining viability for over two weeks. Multi‐modal viability assessment using fluorescence spectroscopy and microscopy (bright‐field and fluorescence) confirmed that droplet culture preserves cell health comparably to bulk methods. The platform supports both single‐cell and few‐cell encapsulation modes, and increasing droplet volume and cell loading density further enhances embryogenesis rates and total embryo yield, respectively. Together, these advances establish a scalable, modular droplet microfluidic platform for high‐throughput embryogenesis analysis and embryo production, offering new opportunities in plant developmental biology, precision breeding, and doubled‐haploid technology.

## Graphical abstract

This work presents a large‐cell‐compatible droplet microfluidic platform that overcomes long‐standing barriers in plant cell culture by enabling high‐fidelity encapsulation, novel multilayer 3D droplet incubation for scalable long‐term culture, and support for the full developmental progression of large plant cells. By integrating spectroscopy and microscopy for label‐free viability tracking, the platform unlocks automated, high‐throughput embryogenesis analysis and scalable embryo production not previously possible in droplet microfluidics.

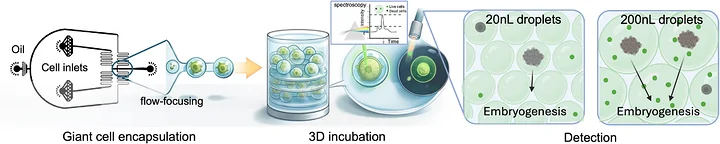

## 1 Introduction

Plant embryogenesis plays a central role in modern breeding, offering a route to rapidly produce genetically uniform lines through doubled haploid (DH) technologies [1, 2]. These completely homozygous lines allow for early [2], precise trait selection [3] and can significantly reduce the time and cost of breeding programs [4]. Traditional breeding often requires 6‐10 generations of selfing or sib‐crossing to obtain highly homozygous lines, whereas DH technology can generate fully homozygous lines within a single generation, dramatically accelerating breeding cycles and genetic gain [5]. Among the available DH techniques, microspore‐derived embryogenesis (MDE)—in which immature pollen grains are reprogrammed under stress to form embryos—is one of the most effective and broadly used [6, 7]. However, this process typically suffers from low efficiency across major crop species [4, 8], often yielding rates of 1–5% in Brassica napus [9, 10], below 1% in barley [11], wheat [12], and maize [8], and near zero in many recalcitrant crops and forest species [4]. Conventional bulk culture systems, such as Petri dishes, remain the gold standard for their simplicity, accessibility, and established protocols. However, they are labor‐intensive, low‐throughput, and challenging to automate, which hinders scalability and consistent embryo production [13]. Additionally, bulk culture fails to capture the heterogeneity of single‐cell responses, limiting our understanding of embryogenic transition [4, 14, 15].

Droplet microfluidics has emerged as a versatile platform for manipulating and studying individual cells in isolated, nanoliter‐scale compartments [16, 17]. These droplets act as miniature bioreactors that offer precise control over the microenvironment, enabling high‐resolution studies of cell behavior, developmental trajectories, and intercellular interactions [18, 19]. The technology supports high‐throughput screening of complex biological systems, including gradient‐based assays involving oxygen, nutrients, or chemical treatments [20, 21], and has been widely adopted for applications such as cell sorting [22, 23] and phenotypic screening in bacterial [24, 25], yeast [26], and mammalian systems [27]. In contrast, its adoption for studies of plant biology and embryogenesis has been limited [10, 28, 29, 30], primarily due to the unique physical characteristics of plant cells. Many are exceptionally large, with maize microspores measuring ∼60–85 µm [31], early multicellular structures reaching ∼100–120 µm, and embryo‐like structures expanding to 200–500 µm [10]. These developmental size increases impose strict constraints on droplet volume, stability, and long‐term culture capability—challenges rarely encountered in typical mammalian or microbial droplet systems. Accommodating such cells requires larger droplets, which tend to be more fragile and prone to instability as droplet volume increases. As a result, only a few studies have explored droplet microfluidics as a platform for encapsulating and culturing large plant cells and microspores [28, 29, 30].

Beyond their use for single‐cell encapsulation, droplet microfluidic systems also hold promise for establishing 3D suspension environments that can overcome many limitations of conventional 2D bulk culture. In Petri dishes, cells quickly settle and form dense layers, restricting nutrient and oxygen exchange and impeding normal development. In contrast, droplet‐based suspension culture can maintain cell spacing, nutrient diffusion, and stable microenvironments, enabling more physiologically relevant growth conditions. However, the potential of such 3D droplet configurations for plant cell culture and embryogenesis has yet to be realized. For DH breeding, a platform capable of supporting long‐term embryogenic development at scale while providing precise control over cell loading and the local microenvironment with single‐cell resolution could enable systematic investigation of how cell density, and cell‐cell interactions influence embryogenesis—parameters that are difficult to isolate in conventional bulk culture, thereby facilitating optimization of embryogenesis conditions and improving embryo production.

We therefore report a droplet microfluidic platform specifically tailored for large plant cells, such as maize microspores and their embryogenic derivatives. The system integrates a tubing‐based rapid loading strategy that reduces sedimentation, robust generation of stable large droplets (20 and 200 nL) for gentle encapsulation, and scalable 3D incubation for long‐term culture. This platform enables controlled, high‐throughput embryogenesis studies while maintaining cell viability for over two weeks. Multi‐modal viability analyses using fluorescence spectroscopy and microscopy confirm that droplet culture preserves cell health comparable to bulk methods. Furthermore, increasing droplet volume and controlled cell loading density markedly enhance embryogenesis rates and total number of embryos generated (total embryo yield). Together, these advances establish a large‐cell‐compatible droplet microfluidic system that bridges high‐throughput single‐cell analysis and scalable 3D culture, laying a foundation for future applications in plant developmental biology, precision breeding, and doubled haploid production.

## 2 Results and Discussion

### 2.1 Platform Overview and Design

**FIGURE 1:**
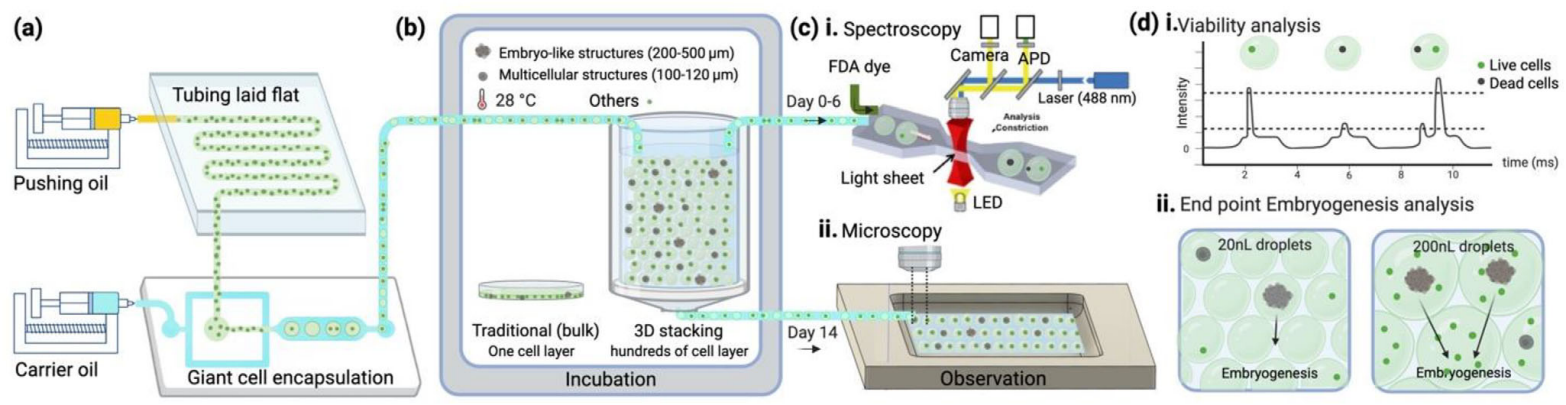
Overview of the scalable droplet microfluidic platform for large plant cell encapsulation and analysis. (a) Giant‐cell encapsulation system: plant cells are pre‐loaded into flat‐laid tubing to minimize sedimentation prior to droplet formation. (b) 3D droplet incubation concept: comparison between conventional 2D bulk culture, where gravity restricts cells to a single layer, and high‐density 3D droplet incubation achieved by stacking multiple droplet layers within a cylindrical reservoir. (c) Detection modalities: i. fluorescence spectroscopy and ii. microscopy. (d) Representative experimental results: i. fluorescence spectroscopy traces showing viability measurements from individual droplets, and ii. microscopy images illustrating embryogenic development across droplets of different sizes and cell densities. Embryogenic cells remain viable after 14 days and can be subsequently planted for further study.

Microspore‐derived embryogenesis (MDE) remains a cornerstone technique for producing doubled haploid (DH) plants, yet it continues to be constrained by low induction efficiency. To address limitations in throughput, culture scalability, and single‐cell resolution, we developed a droplet microfluidic system specifically engineered for the manipulation and long‐term culture of large plant cells. The platform integrates giant cell encapsulation, scalable 3D droplet incubation, and multi‐modal single‐cell analysis, providing a robust framework for stable large‐droplet studies (Figure 1). The workflow begins with rapid cell loading into flat‐laid Tygon tubing, which is then directly connected to the chip inlet (Figure 1a, Figure S1). This tubing‐based loading minimizes sedimentation during sample delivery, ensuring consistent cell concentration and encapsulation fidelity. Within the chip, precisely engineered flow‐focusing geometry and optimized flow rates generate uniform and stable droplets (20 and 200 nL). These droplets are subsequently collected into a cylindrical 3D incubation reservoir for long‐term culture (Figure 1b). Unlike conventional 2D bulk culture, where gravity confines cells to a single layer and limits incubation density, this design enables cells to remain suspended within hundreds of stacked droplet layers, each providing an isolated and stable microenvironment. We have characterized the oil system to enable droplet stacking and high stability. This configuration maintains droplet integrity over extended culture periods and supports the development of multicellular and embryo‐like structures under controlled conditions.

For downstream analysis, the system supports multi‐mode detection (Figure 1c), using i. fluorescence spectroscopy and ii. microscopy. Inline spectroscopic analysis (Figure S2) enables automated, high‐throughput viability assessment, where droplets mixed with fluorescein diacetate (FDA) pass through a light‐sheet fluorescence detector to record individual fluorescence signals. Because FDA is invasive, these droplets are excluded from subsequent embryogenesis monitoring. In parallel, droplets are layered on a customized PDMS reservoir and analyzed using standard microscopy. The detection outputs (Figure 1d), obtained using i. spectroscopy and ii. microscopy, show that both live and dead cells generate fluorescence signals when passing through the light sheet, with live‐cell fluorescence markedly stronger. Complementarily, microscopy provides longitudinal imaging of cell morphology to track embryogenic progression throughout incubation and to recover viable cells for further growth or analysis. Together, these complementary methods provide population‐level viability data and single‐cell morphological information, enabling detailed tracking of embryogenic development across varying droplet sizes and cell densities.

### 2.2 Stable Uniform Droplet Generation and Efficient Loading

A key requirement for high‐throughput embryogenesis screening is the generation of stable, uniform, large‐volume droplets capable of reliably encapsulating the full developmental range of plant cells. Bulk culture optimization revealed that an initial cell concentration of 50 kcells/mL yields the highest embryogenesis rates [32]. To minimize cell–cell interactions and enable single‐cell analysis, we adjusted the inlet cell concentration to maximize single‐cell while minimizing two‐cell droplets (Figure S3). In 20 nL droplets (approximately 330 µm in diameter), single‐cell encapsulation corresponds to an effective cell density of 50 kcells/mL, matching that used in bulk culture. While this approach provided control over encapsulation, consistently producing and maintaining such large droplets with uniform size and stability over long incubation periods posed a significant technical challenge.

**FIGURE 2:**
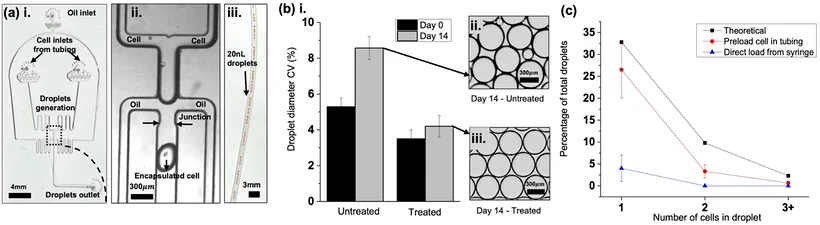
Generation of stable 20 nL droplets and efficient single‐cell encapsulation of maize cells. (a) Microfluidic chip design and operation: i. illustrate the flow‐focusing junction for droplet generation, ii. encapsulation of individual cells, and iii. transport of droplets through Tygon tubing. (b) Effect of chip surface treatment on long‐term droplet stability: i. show that untreated chips (without Aquapel surface treatment) exhibit higher droplet coalescence and increased coefficient of variation (CV) in diameter over 14 days, whereas surface‐treated chips (with Aquapel surface treatment) maintain uniform morphology. CV values were calculated from 200 droplets per condition; experiments were performed in triplicate (n = 3). Insets display representative droplets from ii. untreated and iii. treated chips on Day 14. (c) Comparison of cell‐loading strategies: tubing‐based loading minimizes sedimentation and improves single‐cell occupancy relative to direct syringe loading. The inlet concentration was tuned at 30kcell/mL to maximize single‐cell droplets, maintaining two‐cell droplets below 5%, resulting in λ_*ex*_ ∼ 0.4. Experimental results are compared to theoretical Poisson predictions. Bars represent mean ± SD from four independent experiments (n = 4).

To address this, we systematically evaluated various oil and surfactant formulations for their ability to maintain droplet stability over 14 days. As summarized in Table 1, only HFE‐7500 oil supplemented with 2% FluoroSurfactant consistently yielded droplets that resisted coalescence and preserved uniform size throughout the incubation. The microfluidic chip was engineered with a flow‐focusing geometry (225 µm width, 300 µm height) to avoid cell clogging and enable robust droplet generation (Figure 2a), with optimized oil (4000 µL/hr) and two medium inlets (each 400 µL/hr) flow rates producing highly reproducible 20 nL droplets. After generation, droplets were transferred by Tygon tubing into the 3D reservoir for extended culture. Surface treatments, specifically Aquapel application, were found to be critical for minimizing droplet size variation and maximizing stability. Treated chips exhibited significantly lower coefficients of variation (CV) in droplet diameter compared to untreated chips, a pattern that was maintained after 14 days (Figure 2b).

**TABLE 1:** Evaluation of different oil and surfactant combinations for long‐term droplet stability.

Oil type	Surfactant used	Concentration (%)	Stable Day 0	Stable Day 2	Stable Day 14	Notes
FC‐40	PFO	2%	✗	✗	✗	Droplets were unstable from the beginning and coalesced rapidly
Mineral Oil (AR20)	None	—	✗	✗	✗	No surfactant; droplets formed but coalesced early
HFE‐7500	None	—	✗	✗	✗	Droplets were unstable; rapid coalescence observed
Bio‐Rad Oil for Probes	(pre‐formulated)	—	✓	✓	✗	Initially stable, but droplets began to merge by day 2
HFE‐7500	008‐FluoroSurfactant	2%	✓	✓	✓	Stable droplets over 14 days with appropriate surfactant formulation

To minimize cell loss and improve encapsulation consistency, we developed a tubing‐based, rapid cell loading method. Cells were preloaded into flat‐laid Tygon tubing in under 10 s, minimizing cell sedimentation prior to encapsulation (Figure 1a, Figure S1). Cell recovery was defined as the ratio of the experimentally measured average number of cells per droplet, or experimental average cell occupancy (λ_ex_), to the theoretical average cell occupancy (λ_th_), multiplied by 100. This approach was compared with direct syringe loading with and without magnetic stirring and achieved the highest cell recovery of approximately 60% (Figure S4). To quantitatively evaluate the temporal stability of the tubing‐based loading strategy, the cell concentration at the chip inlet was measured throughout 40 min of continuous droplet generation. No significant change in cell concentration was observed over time (Figure S5a), demonstrating a stable input cell concentration during prolonged operation. To further assess potential cell loss due to adhesion, we quantified cell concentrations before and after passage through the Tygon tubing. No significant difference was observed (Figure S5b), indicating no significant adhesion during loading or droplet generation. Using this optimized loading strategy, we subsequently encapsulated cells in 20 nL droplets at an inlet concentration of 30 kcells/mL, selected to maximize single‐cell encapsulation, and obtained an experimental cell occupancy distribution approaching the theoretical Poisson prediction (Figure 2c), although a noticeable deviation remained due to residual sedimentation loss. The encapsulation process and droplet formation are illustrated in Video S1.

### 2.3 Viability Assessment in Droplet Culture by Microscopy and Spectroscopy

To evaluate the suitability of droplets for maintaining viable maize cells, we first used non‐embryogenic hybrid B73×Mo17 cells, which matched the size of programmed cells but do not undergo embryogenesis, thus enabling cost‐effective analysis. In bulk culture, viability for these cells typically declined from 70% at day 0% to 20% at day 6. In droplets, we aimed to determine whether the microenvironment provided sufficient oxygen and nutrients to sustain viability at comparable levels. While quantitative FDA‐based viability measurements were conducted through day 6 in this section, the extended viability of embryogenic cells (DAS 139/39‐DH) within droplets was further demonstrated by viability analysis through Day 14 and successful embryogenic development at the endpoint, as described in Section 2.4.

**FIGURE 3:**
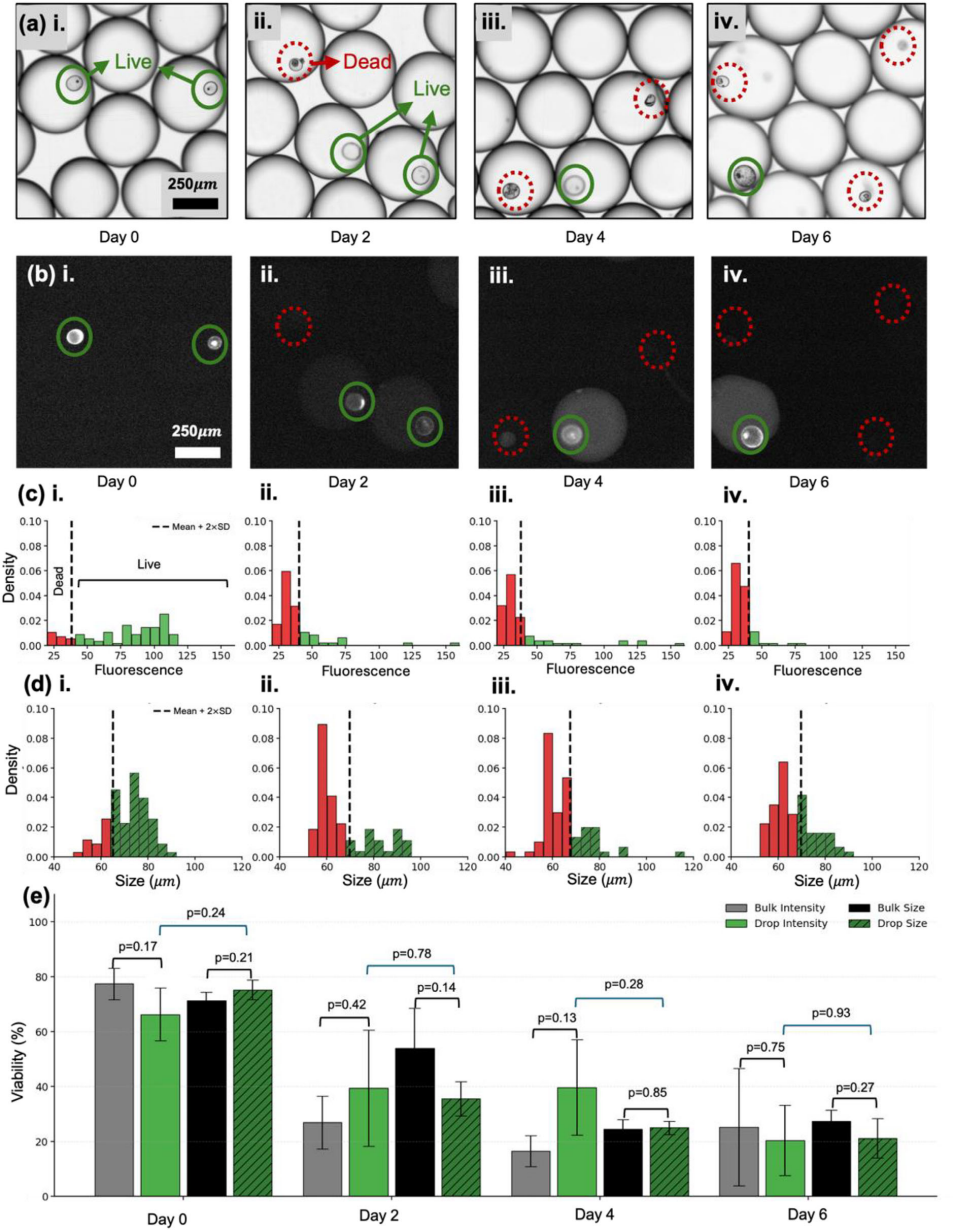
Viability analysis of maize hybrid (B73 × Mo17) cells encapsulated in 20 nL droplets, assessed by FDA fluorescence and cell‐size measurements. (a) Representative bright‐field images of encapsulated cells at i. Day 0, ii. Day 2, iii. Day 4, and iv. Day 6. (b) i‐iv. Corresponding fluorescence images post‐FDA staining: show viable cells (green circles) and dead cells (red dashed circles). (c) i‐iv. Single‐cell fluorescence intensity distributions of encapsulated cells at each time point; viability threshold defined as mean + 2 × SD of the low‐intensity (dead) population (n > 80 cells per group). (d) i‐iv. Cell‐diameter histograms of encapsulated cells from bright‐field images at each time point, used as a noninvasive viability metric with the same thresholding (mean + 2 × SD of the smaller‐diameter, dead population). (e) Comparison of viability rates across time points in bulk culture versus droplets, using both FDA fluorescence and cell‐size analysis. Bars represent mean ± SD from three independent experiments (n = 3). No statistically significant differences were observed between droplet and bulk culture or between the two viability metrics (p > 0.05). λ_ex_ was maintained at ∼ 0.4.

We assessed cell viability in both droplets and bulk cultures using two microscopy methods: fluorescence microscopy with FDA staining (the gold standard) and label‐free bright‐field imaging. We optimized FDA dye incubation time for uniform, robust staining across samples before imaging (Figure S6). Since FDA staining is invasive and is suitable primarily for endpoint measurement, bright‐field microscopy was also employed to enable longitudinal viability measurements without perturbing the cells. As illustrated in Figure 3a, bright‐field images of encapsulated single cells were acquired at days 0, 2, 4, and 6. Viable cells consistently appeared larger and retained unplasmolyzed membranes, while dead cells were smaller and had collapsed morphology. In the corresponding fluorescence images (Figure 3b), living cells were clearly distinguished by high‐intensity (marked in green), while dead cells showed low‐intensity (outlined in dashed red). Viability thresholds were set as the mean plus two standard deviations above the main dead‐cell population, assuming a bimodal distribution. Single‐cell fluorescence intensity (Figure 3c) and cell diameter (Figure 3d) histograms were generated for each time point. For diameter‐based viability analysis, a similar thresholding method was used: on day 0, cell size showed a unimodal distribution, but from day 2 onward, two distinct populations emerged, with viable cells corresponding to the larger fraction (ImageJ workflow is shown in Figure S7).

Maintaining a uniform, single‐layer arrangement of droplets during imaging posed additional challenges, as surface tension in standard mono‐layer reservoirs caused droplets to accumulate at the edges and walls, preventing monolayer formation for high‐content analysis. To address this, we designed a custom reservoir with a curved edge (Figure 1c(ii), Figure S8), which minimized wall accumulation and enabled stable monolayer droplet arrays for reliable microscopy. Comparative analysis of viability rates (Figure 3e) demonstrated no statistically significant differences between droplets and bulk conditions, nor between fluorescence‐ and size‐based assessment methods (p > 0.05). λ_ex_ was maintained at approximately 0.4 throughout the study.

**FIGURE 4:**
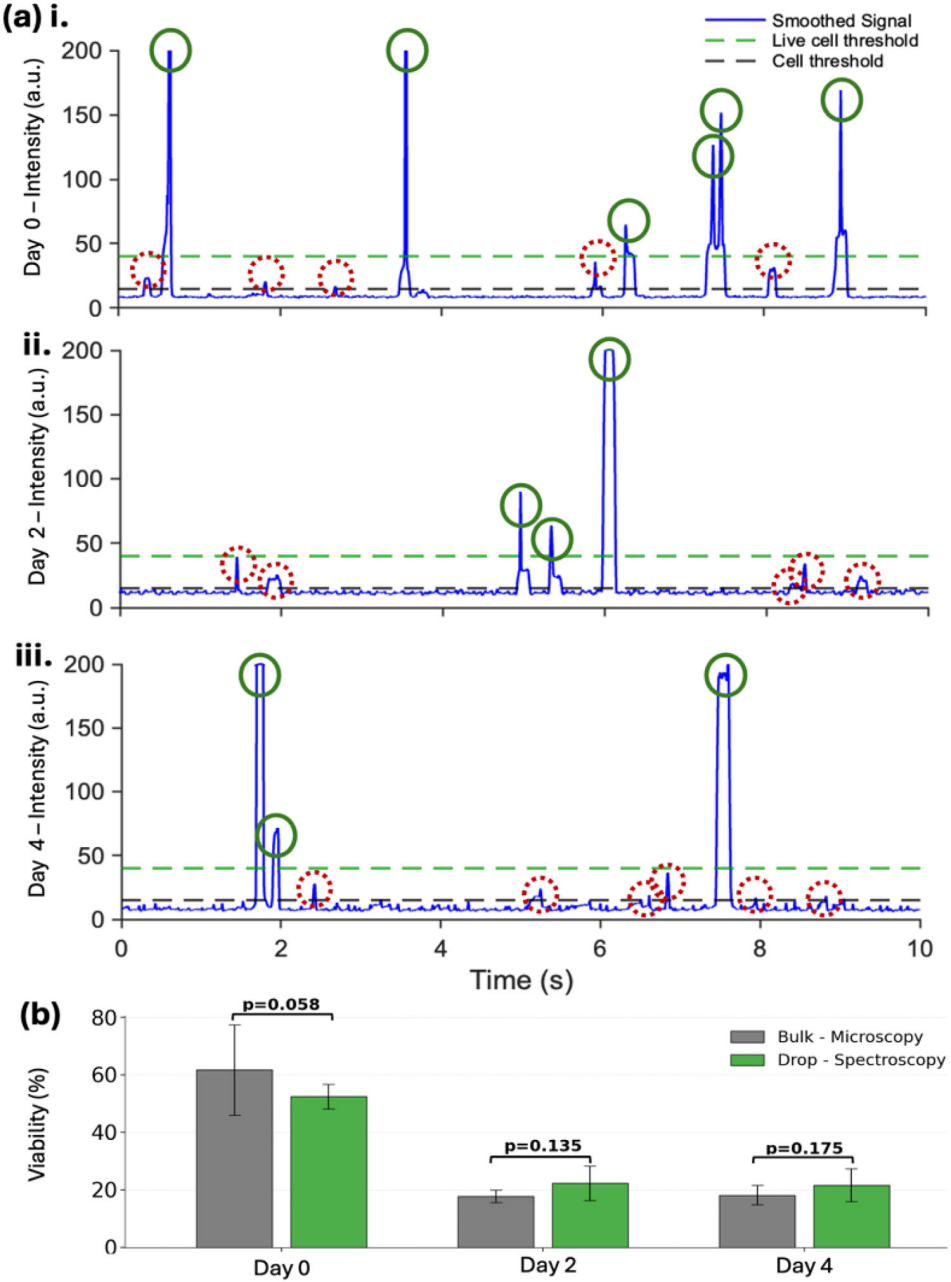
Quantitative assessment of viability in maize B73 × Mo17 cells within 20 nL droplets using light‐sheet fluorescence spectroscopy. (a) Representative spectroscopy traces at i. Day 0, ii. Day 2, and iii. Day 4. Viable cells produce fluorescence peaks exceeding the live‐cell threshold (green dashed line, 40 a.u.), while total cell events exceed the general cell‐detection threshold (gray dashed line, 14 a.u.). Approximately 80 encapsulated cells were analyzed per time point. Initial viability (∼50%) decreased to ∼20% by Days 2 and 4. (b) Comparison of viability measurements obtained by bulk fluorescence microscopy (gray bars) and droplet‐based light‐sheet spectroscopy (green bars). Bars show mean ± SD from three independent experiments (n = 3). No statistically significant differences were observed between droplets and bulk using fluorescence‐based analysis (p > 0.05). λ_ex_ remained at ∼ 0.4.

In parallel with microscopy‐based analysis, droplet cell viability was evaluated using optical spectroscopy, which measures flowing droplets, in contrast to the static droplet imaging used in microscopy. At each time point (days 0, 2, and 4), subpopulations of droplets containing B73×Mo17 cells were collected, stained with FDA, and reinjected through the detection channel for measurement with light‐sheet fluorescence spectroscopy (Figure 1c(i)). Details of the optical setup and components are provided in the Figure S2. This approach enabled rapid, high‐throughput, quantitative viability assessment. Figure 4 summarizes the spectroscopic viability analysis. Representative fluorescence traces (Figure 4a) display individual cell detection over time, with viable cells identified by fluorescence signals exceeding the live‐cell threshold (green dashed line). Initial viability at day 0 was approximately 50%, dropping to ∼20% by day 2 and day 4, in line with trends previously observed by fluorescence microscopy. Quantitative comparison (Figure 4b) confirmed no statistically significant differences between viability values obtained by droplet‐based spectroscopy and the gold standard fluorescence microscopy (n = 3, p > 0.05), supporting the accuracy of the spectroscopic method. Although spectroscopy is more rapid and readily automated, its reliance on FDA makes it an invasive approach, so only a stained subpopulation was analyzed and extrapolated to the remainder of the culture. Nevertheless, the strong agreement with microscopy highlights spectroscopy as a valuable tool for high‐throughput viability screening in droplet systems.

### 2.4 Droplets Enable High‐Throughput 3D Incubation and Embryogenesis

Large plant cells, such as maize microspores (60–85 µm diameter on day 0), present considerable challenges for scalable in vitro culture. In traditional bulk systems, sedimentation occurs rapidly, making it difficult to incubate large numbers of cells without undesired stacking, thus limiting throughput for embryogenesis screening. Unlike conventional 2D culture, where gravity confines cells to thin layers and restricts operational scale, our droplet microfluidic platform individually suspends cells within controlled microenvironments (Figure 1b). By stacking hundreds of droplet layers in a cylindrical reservoir, this approach enables much higher incubation densities, preserves cell spacing, and maintains stable culture conditions for up to 14 days.

**FIGURE 5:**
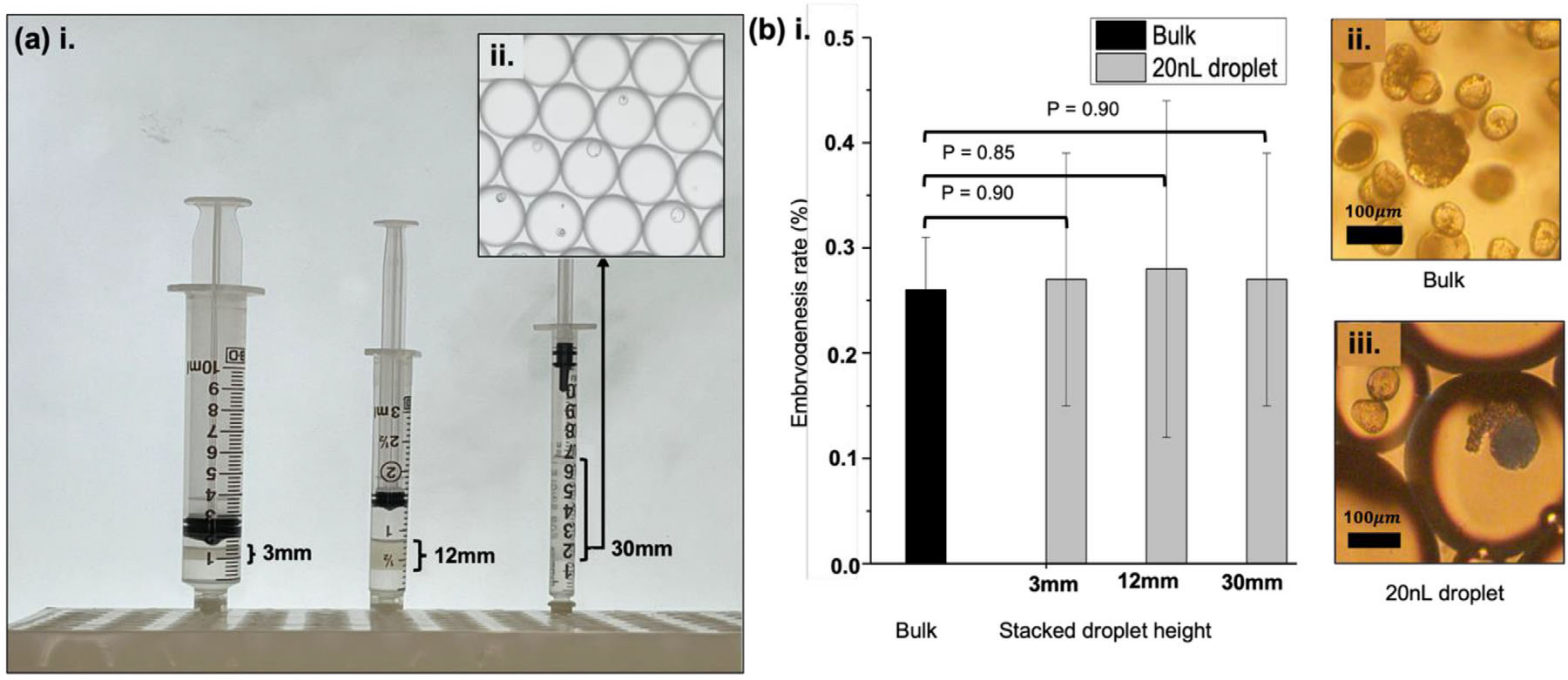
High‐capacity 3D incubation of DAS 139/39‐DH maize cells in droplets with scalable stacking height. (a) Syringe‐based cylindrical reservoirs used to generate adjustable droplet‐stacking heights: i. experimental setup showing 3, 12, and 30 mm stacking heights at a fixed incubation volume of 500 µL, enabling controlled and scalable 3D droplet incubation; ii. bright‐field inset of encapsulated 20 nL droplets after stacking. (b) Embryogenesis assessment across stacking heights: i. embryogenesis rates in bulk culture (black bars) and in 20 nL droplets (gray bars), reported on day 14 as mean ± SD from three independent experiments (n = 3). No statistically significant differences were observed across stacking conditions (p > 0.05). ii. Representative bright‐field image of embryogenic cell clusters in bulk culture; iii. representative image of embryogenesis within individual droplets at Day 14. λ_ex_ was maintained at ∼ 0.2 to keep doublet frequency below 5%.

We first verified that the culture conditions used in the droplet platform were compatible with efficient embryogenesis. To ensure that the addition of carrier oil did not adversely affect embryogenesis rates, bulk cultures with and without carrier oil were compared, revealing no significant differences in embryogenesis rates (Figure S9). Following this compatibility check, cells were encapsulated in 20 nL droplets and incubated in a 5 mL tube, serving as a 3D incubation chamber for 14 days. The overall workflow and key outcomes of the 3D droplet incubation are summarized in Figure 5. Stacking of droplets was demonstrated by using cylindrical syringe reservoirs of various heights 3 mm, 12 mm, and 30 mm (Figure 5a), while maintaining a constant total incubation volume for direct comparison across conditions. Importantly, droplets remained as discrete surfactant‐stabilized emulsion compartments during multilayer incubation, with stable stacking of >100 droplet layers for 20 nL droplets over a 30 mm height without observable widespread coalescence. A quantitative assessment revealed that embryogenesis rates in 20 nL droplets were comparable to those observed in bulk culture, with no significant differences across all stacking heights. Notably, the successful progression to embryogenesis further indicates sustained cellular viability within droplets beyond 14 days. (Figure 5b). ii. Morphological analysis using bright‐field microscopy further demonstrated typical cell cluster formation in bulk and iii. successful embryogenesis within individual droplets after 14 days, with doublet droplets kept below 5% (λ_ex_ = 0.2).

To verify that embryogenesis rate does not increase beyond the 14‐day endpoint in droplets, we next established a method to track the same cells over multiple days reliably. Continuous imaging in an open, planar setup leads to rapid evaporation of oil and medium, causing cell death within hours and preventing long‐term observation. Moreover, droplet drifting and merging further complicate repeated tracking. To address these challenges, we developed a closed Petri‐dish configuration incorporating a PDMS monolayer droplet reservoir and an internal water source for humidity control (Figure S10a,b). This setup minimized evaporation, stabilized droplet positions, and enabled extended imaging of the same droplets across consecutive days (Figure S10c). We first validated that cell viability in fixed droplets remained comparable to conventional bulk culture throughout the imaging period (Figure S10d), confirming that the fixation method itself does not compromise cell viability at least up to 4 days. Using this approach, we confirmed that the number of embryogenic cells remained unchanged at Days 14+2 and 14+4, indicating no further increase in embryogenesis beyond the 14‐day endpoint (Figure S10e). Together, these results validate the 14‐day timeframe for endpoint analysis and demonstrate that our scalable 3D droplet system supports robust embryogenesis with stable viability and consistent droplet organization, providing a reliable platform for high‐throughput parallel screening.

### 2.5 Maximizing Embryogenesis Rates and Throughput

Having shown that 20 nL droplets can match bulk culture embryogenesis rates and that 3D stacking greatly improves incubation scalability, we next investigated increasing droplet volume and cell loading density as strategies to enhance the embryogenesis rate and maximize system throughput, respectively. The relatively low frequency of successful embryogenic events underscores the need to optimize both the rate and the absolute number of embryos produced. As an initial step, we fabricated a microfluidic chip with an expanded flow‐focusing junction (500 µm width and 500 µm height), enabling the reliable generation of 200 nL droplets (Video S2, Figure S11). We first evaluated the suitability of the larger droplets for long‐term culture. Minimal changes in droplet size were observed over a 14‐day incubation period, with a coefficient of variation (CV) of approximately 2% and 4% at Day 1 and Day 14, respectively (Figure S12), confirming the stability of the 200 nL droplet format during extended culture. We then compared the viability of non‐embryogenic B73×Mo17 cells cultured in 20 nL droplets, 200 nL droplets, and conventional bulk culture over 6 days using FDA staining. No significant differences in viability were observed between the two droplet formats at any evaluated time point (Figure S13a), and viability in both remained comparable to or higher than that in bulk culture. Similarly, embryogenic DAS 139/39‐DH cells exhibited no significant differences in viability between the 20 and 200 nL droplet formats during 14 days of incubation (Figure S13b), with both formats maintaining viability comparable to or exceeding that of bulk culture. Together, these results demonstrate that increasing droplet volume from 20 to 200 nL preserves long‐term cell viability, including embryogenic cell survival prior to embryo formation.

**FIGURE 6:**
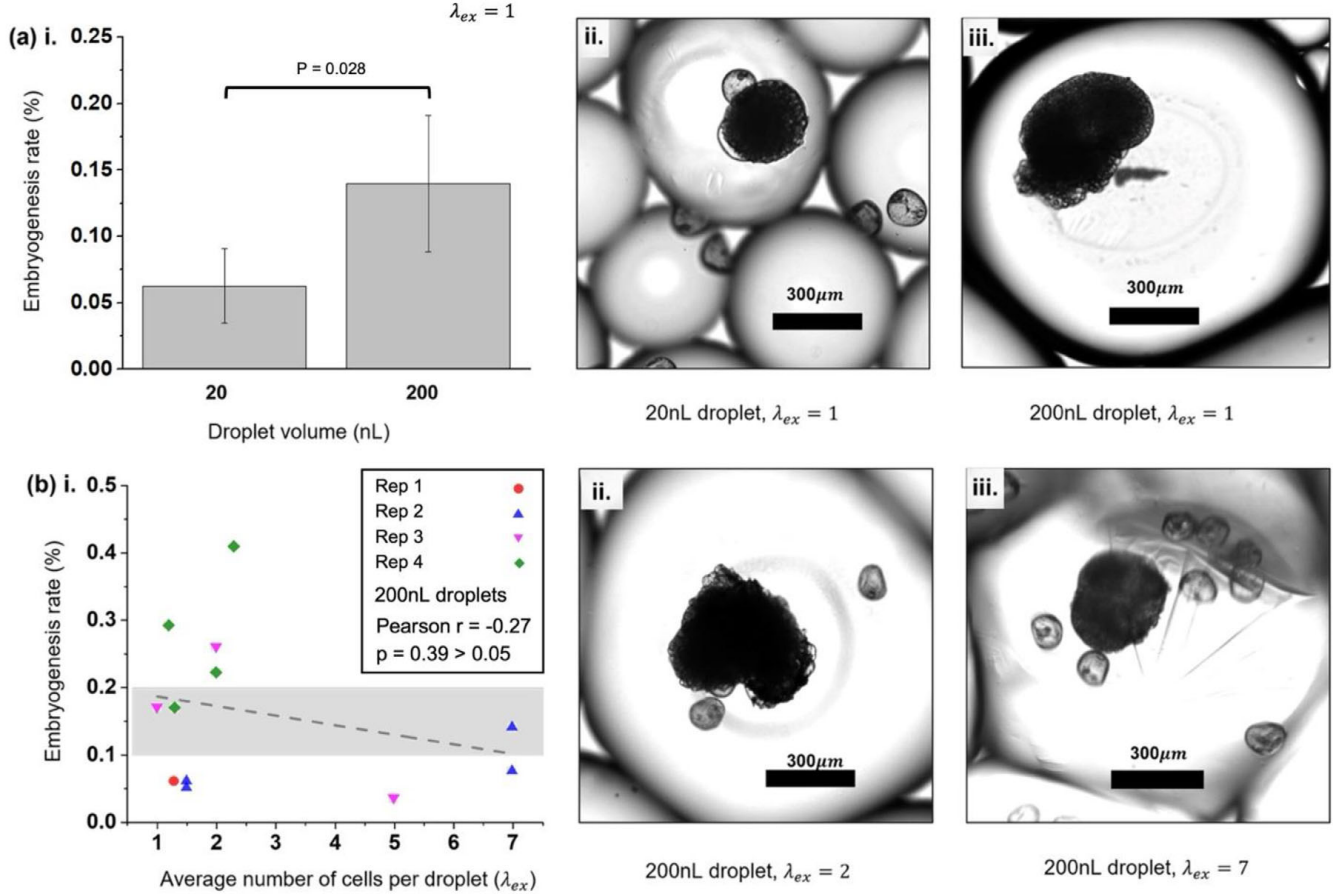
Impact of droplet volume and cell loading density on embryogenesis rate in encapsulated DAS 139/39‐DH maize cells. (a) i. Embryogenesis rates in 20 nL and 200 nL droplets at fixed experimental average number of cells per droplet (λ_ex_ = 1); larger droplets significantly increase embryogenesis rates (n = 4, p < 0.05). ii. Representative bright‐field images of embryogenic clusters formed in 20 nL and iii. 200 nL droplets, both at λ_ex_= 1, with enhanced cluster formation in 200 nL droplets. (b) i. Relationship between embryogenesis rate and experimentally measured average number of cells per droplet in 200 nL droplets, demonstrating throughput scalability (n = 4 independent experiments per group). ii. Bright‐field images of embryogenic clusters in 200 nL droplets at low (λ_ex_= 2) and iii. high (λ_ex_= 7) average number of cells per droplet. All images were taken at Day 14 of the incubation.

Having established the compatibility of the 200 nL droplets for long‐term culture, we next examined whether increasing droplet volume from 20 to 200 nL could improve embryogenesis. Using these droplets at a constant average number of cells per droplet, embryogenesis rates increased in 200 nL droplets (Figure 6a). Bright‐field images acquired on Day 14 further revealed that larger droplets supported continued growth of embryonic structures, which often exceeded 300 µm in diameter. This improvement is likely due to increased nutrient availability in larger droplets. Importantly, despite this substantial embryogenic growth, approximately 85% of droplets containing embryogenic structures remained stable after 14 days of incubation while the remaining 15% of them exhibited size increases consistent with potential coalescence (Figure S14).

To further increase embryo yield and overall system throughput, we next investigated the effect of cell loading in 200 nL droplets. Before evaluating embryogenesis, we first assessed whether increasing cell loading influenced cell viability. Non‐embryogenic B73×Mo17 cells and embryogenic DAS 139/39‐DH cells were encapsulated at inlet cell concentrations of 5000, 10 000, and 50 000 cells/mL, corresponding to theoretical average number of cells per droplet of λ_th_ = 1, 2, and 10, respectively (Figure S15). Cell viability was assessed by FDA staining. In both cell types, higher cell occupancies maintained viability comparable to conventional bulk culture, whereas lower occupancies showed a trend toward reduced viability over time. These findings suggest that lower cell loading may negatively affect cell survival, potentially due to diminished cell‐cell interactions, although further mechanistic studies are needed to confirm this hypothesis.

We next evaluated the effect of cell loading on embryogenesis. For these experiments, cells were encapsulated at inlet cell concentrations of 10 000, 20 000, and 50 000 cells/mL, corresponding to theoretical average cell occupancies of λ_th_ = 2, 4, and 10, respectively. Due to partial cell sedimentation during loading, the experimentally measured occupancies were lower (λ_ex_ ≈ 1–7), over which the embryogenesis rate remained relatively unchanged (Figure 6b). The embryogenesis rate showed a weak negative correlation with λ_ex_ that was not statistically significant (Pearson r = −0.27, p = 0.39), suggesting a weak relationship between cell loading and the probability of embryogenic development under the conditions examined. In contrast, the total number of embryos generated increased proportionally with λ_ex_, reflecting the greater number of encapsulated cells per droplet while preserving cell viability and developmental potential.

## 3 Conclusions

In this work, we introduced a droplet microfluidic platform specifically tailored to the unique size and developmental progression of large plant cells, enabling reliable encapsulation, scalable long‐term 3D culture, and multi‐timepoint analysis of microspore‐derived maize embryogenesis. By combining stable 20 and 200 nL droplet generation, optimized flow conditions, and surface‐treated microchannels, we overcame the technical challenges of encapsulating fragile, sedimentation‐prone cells. We developed a tubing‐based loading strategy to minimize cell loss and achieved high encapsulation fidelity with Poisson‐predicted values.

For droplet incubation, our approach offers a distinct advantage over conventional 2D culture: rather than being restricted to a thin layer by gravity, our platform enables droplets to be stacked in hundreds of layers within cylindrical reservoirs, dramatically increasing incubation density while preserving cell spacing and stable microenvironments for extended culture for up to 14 days. Moreover, our system integrates both microscopy (bright‐field and fluorescence) and fluorescence spectroscopy‐based viability assessment of cells encapsulated in droplets, with results validated against standard 2D culture. The establishment of label‐free bright‐field size analysis as a robust, noninvasive method further enables longitudinal tracking and ensures that viable cells remain suitable for continued growth or planting. Moreover, increasing droplet volume and cell loading density were shown to enhance embryogenesis rates and expand analytical throughput, respectively, allowing scalable, parallel embryo production.

Compared with previously reported droplet microfluidic systems, which primarily focused on smaller cells such as *Chlamydomonas reinhardtii* and plant protoplasts encapsulated in picoliter‐ to low‐nanoliter‐volume droplets [28], the present platform was designed for substantially larger maize microspores (65–85 µm) and embryo‐like structures. While recent studies have demonstrated embryogenic development within droplets and long‐term culture in larger droplet formats [10], these systems primarily utilized tubing‐based incubation. In contrast, our platform employs a high‐density 3D incubation format that facilitates droplet storage, sample retrieval, and scalable long‐term culture of large maize microspores (65–85 µm). Together, these innovations establish a scalable, modular, and automatable platform capable of supporting the full developmental range of large plant cells through high‐density 3D incubation, multimodal viability assessment, and scalable embryo production—capabilities that remain largely unexplored in droplet microfluidics for plant embryogenesis.

Despite these advances, several opportunities remain for further development of the platform. First, while we achieved stable droplet generation and cell viability for up to 14 days, longer‐term culture studies in 200 nL droplets may reveal insights into later stages of embryogenesis and enable recovery of fully regenerated plantlets. Second, we anticipate integrating this platform with on‐chip hormone or stressor delivery to dissect the temporal dynamics of embryogenic reprogramming at single‐cell resolution. Third, the relationship between cell loading density, cell viability, and embryogenic development warrants further mechanistic investigation. Future studies combining controlled cell loading with molecular and transcriptomic analyses could elucidate the role of local cell‐cell interactions in regulating these processes and further optimize droplet‐based embryogenesis. Fourth, automation of image‐based analysis and droplet sorting could further increase throughput and enable real‐time feedback for condition screening. Another promising direction is the development of noninvasive detection of cells using autofluorescence signals acquired by spectroscopy, which could enable truly inline, high‐throughput, and label‐free viability analysis. Finally, expanding the platform to accommodate diverse plant species and cell types, including recalcitrant or non‐model crops, would broaden its applicability. With these advancements, the presented large‐cell‐compatible droplet microfluidic system has the potential to serve as a foundational tool for plant synthetic biology, precision breeding, and next‐generation doubled haploid production.

## 4 Methods

### 4.1 Plant Material and Microspore Isolation

Maize microspore donor plants B73xMo17 and DAS 139/39‐DH were grown in greenhouses under a 16 h photoperiod, well watered, and fertilized with 150 ppm 17‐4‐17 fertilizer as needed. Tassels of B73xMo17 and DAS 139/39‐DH were harvested just before emergence from the leaf whorl and when a majority of the microspores are at the late uni‐nucleate to early bi‐nucleate developmental stage. The tassels are then cold treated for at least 3 days at 10°C in the dark. To isolate the microspores, spikelets were removed from a tassel, surface sterilized in a 25% bleach solution for 15 min, and rinsed 4 times with sterile distilled water. Microspores were released from anthers in isolation medium as described by Gaillard et al. [33]. using a Waring blender with an MC3 250 mL mini‐sample container. The slurry is then passed through a set of stainless‐steel sieves with a mesh size of 125–180 µm to remove large debris and 20–25 µm to retain microspores. Microspores are then further purified through a series of washes and density gradients similar to Gaillard et al. [33].

### 4.2 Microfluidic Chip Design and Fabrication

The single‐layer microfluidic chip was designed in AutoCAD 2021 (Autodesk, San Rafael, CA, USA) to produce monodisperse droplets of ∼20 nL or ∼200 nL using a flow‐focusing geometry with two aqueous inlets, one oil inlet, and a single outlet. Channel dimensions and junction geometry were optimized to accommodate large maize microspores and maintain droplet stability over long‐term incubation. The finalized design was exported as a photomask and printed on high‐precision transparencies at 20,000 dpi (CAD/Art Services Inc., Bandon, OR, USA). The master mold was fabricated on a 4‐inch silicon wafer (Polishing Corporation of America, Santa Clara, CA, USA) using standard photolithography. SU‐8 3050 negative photoresist (MicroChem Corp., Newton, MA, USA) was spin‐coated, patterned by UV exposure through the photomask, and developed according to the manufacturer's instructions. Two chip designs were fabricated. For the 20 nL droplet generation chip (junction width: 225 µm, height: 300 µm), a uniform photoresist thickness of 300 µm across the wafer was achieved by three sequential spin‐coating and soft‐baking cycles. Each spin‐coating step consisted of 500 rpm with an acceleration of 100 rpm s^−^^1^ for 20 s, followed by 1000 rpm with an acceleration of 300 rpm s^−^^1^ for 60 s, and was followed by a soft‐bake at 65°C for 5 min and 95°C for 45 min before the next layer was applied. The 200 nL droplet generation chip (junction width: 500 µm, height: 500 µm) was fabricated using the same three‐layer process and spin parameters, producing a total photoresist thickness of 500 µm. Soft lithography was applied to fabricate the PDMS device. SYLGARD 184 silicone elastomer (Dow Corning, Midland, MI, USA) was mixed at a 10:1 base‐to‐curing agent ratio, poured over the master mold, degassed under vacuum, and cured at 80°C for 1 h. The cured PDMS slab was peeled from the mold, and inlet and outlet ports were punched. The device was then bonded to a glass slide using oxygen plasma treatment (42 W, 500 mTorr, 45 s). Chips were rendered hydrophobic as part of the device preparation process by flushing the entire microfluidic chip with Aquapel (PGW Auto Glass, LLC, PA, USA), followed by air purging to remove Aquapel reagent and baking at 65°C for 1 h before use. The difference with and without Aquapel treatment was assessed by monitoring the droplet images using a hemocytometer (Hausser Scientific, Horsham, PA, USA) under an inverted fluorescence microscope (Olympus IX71, Tokyo, Japan).

### 4.3 Customized 2D Reservoir Design and Fabrication

The reservoir mold was designed using Autodesk Fusion 360 (Autodesk Inc., educational license) to accommodate the formation of a layer of droplets and facilitate efficient imaging. The reservoir geometry featured a “U”‐shaped bottom profile to avoid stacking droplets over each other near the edges and minimize dead space (Figure S8). Design files were exported and fabricated using a Bambu Lab X1–Carbon 3D printer (Bambu Lab, Shenzhen, China) with standard PLA filament, following manufacturer‐recommended parameters. The printed mold was embedded in uncured polydimethylsiloxane (PDMS) (SYLGARD 184, Dow Corning, Midland, MI, USA) at a thickness of 1–2 cm and cured at 80 °C for 1 h. After curing, the 3D printed template was carefully removed to yield the PDMS reservoir structure. The PDMS reservoir was bonded to thick glass slides (75 x 50 mm; Ted Pella, Inc., Redding, CA, USA) via oxygen plasma treatment (42 W, 500 mTorr, 45 s) to ensure leak‐free adhesion and device integrity. All devices were rinsed and baked at 65 °C for 1 h prior to use.

### 4.4 Theoretical and Experimental Poisson Distribution of Encapsulated Cells

To quantify encapsulation efficiency, we compared the theoretical Poisson distribution with the experimentally observed distribution of cell numbers per droplet. The theoretical probability *P_th_* (*X*) of finding *X* cells in a droplet was calculated using the Poisson formula (Equation 1):

(1) $$P_{th}\left(X\right)=\frac{\lambda_{th}^{x}e^{-\lambda_{th}}}{X!}$$

where λ_th_ is the theoretical average number of cells per droplet or cell occupancy, and was obtained as the product of cell inlet concentration (cells/mL) and droplet volume (mL). However, due to cell sedimentation in the loading tubing and imperfect delivery to the microfluidic junction, the actual concentration at the droplet generation site—and thus experimental average number of cells per droplet (λ_ex_)—was lower than the theoretical estimate. To determine the λ_ex_, images of at least 200 droplets were acquired, and the total number of encapsulated cells was counted. The λ_ex_ was calculated by dividing the total number of encapsulated cells by the total number of droplets counted. The experimental distributions were generated by categorizing droplets according to the number of cells contained (0, 1, 2, etc.) and calculating the observed frequencies, which were subsequently compared with the theoretical prediction. The corresponding average cell density within the droplets was calculated by dividing the experimentally measured average number of cells per droplet (λ_ex_) by the droplet volume.

### 4.5 Prevention of Cell Sedimentation During Loading

To maintain a constant inlet concentration of large microspores, cells were preloaded into Tygon microbore tubing (0.02‐inch ID, 0.06‐inch OD; Cole‐Parmer, Vernon Hills, IL, USA) prior to encapsulation (Figure S1). The tubing length was selected according to the desired loading volume. Upstream, the tubing was connected to a syringe pump containing fluorinated oil FC‐40 (3 M, Two Harbors, MN, USA), while the downstream end was connected directly to the chip inlet. During operation, the tubing was positioned at the same horizontal height as the microfluidic chip to minimize gravitational settling. The final 10 cm of the cell‐containing tubing was excluded from droplet generation because gradual cell sedimentation during droplet generation resulted in an increased cell concentration in this terminal portion. This configuration reduced sedimentation and achieved approximately 60% of the theoretical Poisson‐predicted single‐cell loading rate.

Cell adhesion during tubing loading was evaluated by transferring a cell suspension from a 5 mL conical tube through 2 m of Tygon tubing using the standard cell loading procedure. FC40 was used as the pushing oil to displace the aqueous cell suspension into a 1.5 mL centrifuge tube. Loading was continued until the FC40–aqueous interface reached the tubing outlet, ensuring complete transfer of the cell suspension through the tubing. Cell concentration before and after tubing loading was quantified by counting 3 µL aliquots using a hemocytometer.

### 4.6 Droplet encapsulation and Stability Screening

Once the cells (B73xMo17 or DAS 139/39‐DH) are loaded into Tygon microbore tubing (0.02‐inch ID, 0.06‐inch OD; Cole‐Parmer, Vernon Hills, IL, USA), droplets were generated by co‐flowing the one aqueous microspore suspension and two oil phases through the flow‐focusing junction of the microfluidic chip, resulting in discrete surfactant‐stabilized droplets encapsulating individual or multiple plant cells depending on the experimental loading condition. The generated droplets were then collected in a tube for further analysis and downstream incubation. To determine the optimal long‐term stability conditions, multiple oil–surfactant formulations were screened over a 14‐day incubation at 28°C. Formulations tested included FC‐40 with 2% (w/w) perfluoro‐1‐octanol (PFO; Sigma–Aldrich, St. Louis, MO, USA), mineral oil (AR20; Sigma–Aldrich, St. Louis, MO, USA) without surfactant, HFE‐7500 (3 M, Maplewood, MN, USA) without surfactant, Bio‐Rad Oil for Probes (Bio‐Rad Laboratories, Hercules, CA, USA; pre‐formulated), and HFE‐7500 containing 2% (w/w) 008‐FluoroSurfactant (RAN Biotechnologies, Beverly, MA, USA). Among all candidates, HFE‐7500 with 2% 008‐FluoroSurfactant produced the most stable droplets, remaining intact throughout the 14‐day test period. Using this formulation, flow rates were optimized for droplet size control: for ∼20 nL droplets, the aqueous and oil flow rates were set to 800 and 4000 µL h^−^^1^, respectively (Video S1), while larger ∼200 nL droplets were produced by adjusting the aqueous and oil flow rates to 2000 and 4000 µL h^−^^1^, respectively (Video S2).

Droplet stability for 20 nL droplets was assessed at Days 0, 7, and 14 using a hemocytometer (BLAUBRAND, BR719820, Wertheim, Germany) under an upright fluorescence microscope (Olympus BX51, Tokyo, Japan), as shown in Figure 2b. For 200 nL droplets, an open PDMS reservoir was used to prevent merging of the larger droplets during imaging (Figure S12). Droplet images were segmented and analyzed using ImageJ with the “Analyze Particles” tool. To evaluate droplet stability during embryogenic expansion, the diameter distribution of non‐embryogenic droplets was used as a reference. Droplets with diameters exceeding three standard deviations above the mean diameter of the non‐embryogenic population, corresponding approximately to twice the initial droplet volume, were classified as merged (Figure S14).

### 4.7 6‐well Plate for Bulk Culture

Bulk culture experiments were run in parallel in a typical 6‐well plate setup (Corning Inc., Corning, NY, USA) to serve as a reference and positive control. One well was seeded with 2 mL of cell suspension at a concentration of 50,000 cells per mL. To maintain humidity and minimize evaporation, two adjacent wells were filled with sterile deionized water. The plate was sealed with Parafilm (Bemis Company, Inc., Neenah, WI, USA) to prevent leakage and ensure stable culture conditions. Following 14 days of incubation, embryo‐like structures were quantified using an inverted fluorescence microscope (Olympus IX71, Tokyo, Japan); the inverted configuration was chosen due to the limited working distance of objectives in upright microscopes, which are incompatible with the well plate format.

### 4.8 Assessment of Cell Viability Using Microscopy

Cell viability in both bulk suspension and microfluidic droplets was assessed using fluorescein diacetate [34] (FDA; Sigma–Aldrich, St. Louis, MO, USA) and compared with size analysis. Since programmed maize cells for embryogenesis are labor‐intensive and expensive, first we used hybrid B73×Mo17 maize cells, which have no embryogenesis potential but are of similar size and exhibit extended viability in bulk culture. On day 0, a significant population of both bulk and encapsulated microspores was generated, and at each subsequent time point (days 0, 2, 4, and 6), a subpopulation of more than 80 cells was collected for viability analysis to ensure reproducibility (Figure S16). FDA dye was added directly to the generated droplets in oil, relying on its ability to diffuse through the fluorinated oil phase into the aqueous droplets over time. A 4 mg mL^−^^1^ FDA stock solution in acetone was diluted 1:100 in dH_2_O to prepare a fresh working solution, which was then further diluted 1:100 prior to application to either the microspore suspension (bulk) or the cell‐containing droplets in oil, yielding a final FDA concentration of 0.4 µg mL^−^^1^ (∼1 µM). To ensure effective and uniform staining, given the limited solubility and slow diffusion of FDA in the oil phase, droplets were incubated with FDA for 70 min at room temperature prior to imaging (Figure S6). For experiments involving 200 nL droplets, a 10‐fold higher FDA concentration was used for all conditions to ensure uniform staining of encapsulated cells.

Viability measurements were performed in a customized PDMS observation chamber using an upright fluorescence microscope equipped with a GFP filter set. Viable cells in bright field images were identified by circular shape and large size, whereas non‐viable cells appeared plasmolyzed and shrunken. Similarly, in fluorescence images, viable cells appeared with bright intracellular fluorescence, whereas non‐viable cells appeared non‐fluorescent. Image processing was performed with ImageJ, using bright‐field images for cell segmentation to create individual cell masks, and the ROI Manager was applied to quantify mean fluorescence intensity and cell size for each cell in the corresponding fluorescence image (Figure S7). Fluorescence intensity values were plotted as histograms for fluorescence viability analysis, and cell size histograms were used for size‐based viability assessment. Live and dead cell populations were separated using a threshold defined as the mean plus two standard deviations (mean + 2SD) of the dead cell population, and cell viability percentage was calculated as the percentage of live cells among the total counted cells. Comparative bar graphs for viability analysis under the two methods were generated and visualized in Python.

### 4.9 Assessment of Cell Viability Using Spectroscopy

Cell viability in droplets was additionally assessed by optical spectroscopy. On day 0, a significant population of droplets containing cells was generated, and at each subsequent time point (days 0, 2, and 4), a subpopulation was collected for analysis (cell viability assessments were limited to day 4 because sample shipment during the summer resulted in lower viability on day 0). FDA dye was added to each sample and incubated for 70 min at room temperature as described above. Droplets were then reinjected into the PDMS chip from the outlet port (i.e., in reverse direction to droplet generation), with oil inlets closed using dispenser needles (McMaster‐Carr, stainless steel, 23 gauge, 1.5′ length). The light sheet was oriented perpendicular to the microchannel at its smallest cross‐section, and droplets were preloaded into Tygon tubing (0.02‐inch ID, 0.06‐inch OD; Cole‐Parmer), then reinjected at a constant flow rate of 800 µL/hr. Spectroscopic data were collected continuously and processed using MATLAB; raw data were smoothed using a window size of 100, and cell counting was performed using two‐step intensity thresholding, with the first threshold identifying all cells (live and dead) and the second threshold classifying live cells based on fluorescence signal. Viability was calculated as the percentage of live cells among the total counted cells.

### 4.10 Light Sheet Fluorescence Detection Setup

A custom light sheet fluorescence detection system was used to measure cell viability in droplets on the microfluidic chip based on our previously point laser‐induced fluorescence design [35] with modifications to form light sheet (Figure S2) [36]. Three lasers (OBIS 488, OBIS 552, and OBIS 640; Coherent) served as excitation sources and were combined using a laser combiner, with the beam profile further homogenized by a Gaussian beam corrector. The collimated laser beam had a diameter of approximately 3 mm after neutral‐density filtering and Gaussian beam correction and was subsequently passed through a plano‐convex cylindrical lens (*f* = 150 mm, Ø1″, Thorlabs) to form the light sheet. The resulting beam was directed onto a quad‐band dichroic mirror (ZT405/488/561/640rpc, Chroma) and focused onto the detection region of the PDMS chip using a 4× objective (PLN 4X, NA 0.10, Olympus). Based on geometrical‐optics calculations using the thin‐lens equation, the lateral width of the light sheet at the interrogation region was estimated to be approximately 900 µm, consistent with the experimentally measured width. This width was substantially larger than the 225 µm microchannel width at the interrogation region, ensuring that the entire channel remained within the central region of the light‐sheet profile and providing sufficient room for lateral alignment. The light‐sheet thickness was theoretically estimated using the Gaussian beam waist relation, assuming an ideal Gaussian beam (*M* ^2^ = 1), resulting in a full 1/*e* ^2^ thickness of approximately 12.2 µm. As droplets containing cells traversed the microfluidic detection window, excitation occurred in real time; the emitted fluorescence was collected by the same objective and subsequently separated into spectral channels using a series of dichroic mirrors (FF552‐Di02‐25x36, FF660‐Di02‐25x36, ZT405/488/561/640rpc, Chroma). Fluorescence signals in each channel were detected by avalanche photodiode (APD) detectors (SPCM‐AQRH‐10, Excelitas). For all FDA dye measurements, Channel 1 was selected. Analog detector outputs were digitized using a National Instruments analog‐to‐digital converter and recorded for subsequent analysis.

### 4.11 Three‐Dimensional (3D) Droplet Incubation and Embryogenesis Assay

High‐capacity droplet culture was performed in cylindrical syringe reservoirs to evaluate “foam factor” effects on incubation density. Reservoirs were incubated at 28°C for up to 14 days. Embryogenesis rates were compared between bulk Petri dish cultures and droplets of different volumes (20 nL vs. 200 nL) and different cell loadings (λ_ex_ = 1–7). Developmental progression was documented using bright‐field microscopy, and embryogenesis was scored based on characteristic morphological features. Cell clusters were classified as embryogenic if they exhibited a visible embryoid structure, had at least one dimension larger than 120 µm, and showed clear evidence of membrane breakdown.

Embryogenesis rate was calculated as follows: First, λ_ex_ was determined as described above. The total number of droplets was obtained by dividing the overall sample volume by the individual droplet volume. The estimated total number of encapsulated cells was then calculated by multiplying λ_ex_ by the total droplet count. Finally, embryogenesis rate was defined as the number of observed embryogenic cells divided by the estimated total number of cells.

### 4.12 Statistical Analysis

Unless otherwise stated, experiments were performed in triplicate (n = 3). Quantitative results are presented as mean ± standard deviation (SD). Statistical comparisons between groups were conducted using the two‐sample unpaired t‐test with Welch's correction to account for unequal variances. Results with p < 0.05 were considered statistically significant. All relevant analyses and calculations were performed using Python and MATLAB.

### 4.13 Graphing and Figure Preparation

Graphs and plots were generated using OriginLab (OriginLab Corporation, Northampton, MA, USA) and Python for quantitative results. Schematic illustrations were designed using BioRender and Fusion 360 (Autodesk Inc., educational license) to support experimental visualization.

## Author Contributions

A.M. and F.S. contributed equally to this work. A.M. and F.S. conceived the project and designed the microfluidic platform, including chip fabrication, oil selection, droplet stability optimization, and cell loading strategies. A.M. performed microscopic imaging, viability analyses, and data analysis. A.M. and L.W. conducted spectroscopic viability measurements, and A.M. and T.J. carried out the 3D droplet stacking experiments. A.M. and G.D. performed additional experiments and data analysis during manuscript revision. F.S. assisted in preparing the initial draft, including the Materials and Methods and Conclusions sections. A.M. completed the manuscript and wrote the Abstract, Introduction, Results, and Supporting Information. E.C., Y.B., D.S., K.H., and H.G. provided biological materials and technical support. T.‐H.W. and K.H. supervised the project. This work was funded by Corteva Agriscience. All authors discussed the results and approved the final manuscript.

## Conflicts of Interest

The authors declare no conflicts of interest.

## Supporting information




**Supporting File 1**: advs77936‐sup‐0001‐SuppMat.docx.

**Supporting File 2**: advs77936‐sup‐0002‐VideoS1.mp4.

**Supporting File 3**: advs77936‐sup‐0003‐VideoS2.mp4.

## Data Availability

The data that support the findings of this study are available from the corresponding author upon reasonable request.
