# Supplementary material for: A Scalable Droplet Microfluidic Platform That Overcomes Large‐Cell Constraints for Long‐Term Plant Cell Culture and Embryogenesis Analysis
Source: Adv Sci (Weinh). 2026 Sep 27:e77936. Online ahead of print. doi: 10.1002/advs.77936 (PMC13616392; doi:10.1002/advs.77936)
Supplement: Supplementary file 1 [file ADVS-9999-e77936-s002.docx]

**Supporting Information**

**A scalable droplet microfluidic platform that overcomes large-cell limitations for long-term plant cell culture and embryogenesis analysis**

*Arman Mirmiran^§^, Fangchi Shao^§^, Gregory Datto, Tathansh Joshi, Elizabeth Chatt, Yashar Bashirzadeh, Dan Stessman, Lai Wei, Kuangwen Hsieh, Hena Guo, Tza-Huei Wang**

**Figure S1**. **Stepwise procedure for loading microspores into tubing to prevent cell sedimentation prior to encapsulation.** **(a)** The microspore suspension is vortexed at 1000 rpm to ensure uniform cell distribution. **(b)** Tubing is cut to accommodate the desired sample volume (e.g., ~1 m tubing per 200 μL), and fitted with a needle and syringe. **(c)** The microspore sample is aspirated into the tubing; the tubing is then positioned to allow the sample to exit at the interface, removing air, before being trimmed. **(d)** The tubing containing the microspores and incubation medium is connected to a syringe pump-driven tubing filled with FC40 oil (“pushing oil”), with the other end connected to the chip inlet. This approach maintains a consistent cell concentration at the chip inlet, effectively mitigating sedimentation and supporting reliable giant cell encapsulation.

**Figure S2. Schematic of the light-sheet fluorescence spectroscopy setup and excitation geometry. (a)** Optical configuration showing the laser source and combiner, neutral-density (ND) filters and Gaussian correction, cylindrical lens for light-sheet formation, dichroic mirrors, beam splitter, imaging optics, and avalanche photodiode (APD) detectors for fluorescence detection. **(b)** Top view of the microchannel and excitation light sheet, illustrating the ~12.2 µm light-sheet thickness and ~900 µm lateral width at the interrogation region. The light sheet is oriented orthogonally to the droplet flow direction and is substantially wider than the 225 µm microchannel, ensuring coverage of the full channel width and relatively uniform excitation across the interrogation region. **(c)** Side view of the detection region, showing the PDMS microchannel on the glass substrate and the 4× objective. The excitation light sheet intersects the microchannel at the interrogation region, allowing droplets and encapsulated cells to traverse the excitation plane during detection.

**Figure S3. Optimization of cell concentration for single-cell encapsulation in 20 nL droplets**. The fraction of droplets containing 0, 1, 2, or ≥3 cells was measured as a function of input cell concentration (20, 30, 50, and 80 kcell/mL). The experiment was designed to maximize single-cell encapsulation while maintaining a throughput in which fewer than 5% of droplets contain doublets. At a concentration of 30 kcell/mL, more than 20% of droplets contained a single cell, with less than 5% doublets, indicating optimal conditions for maximized single-cell occupancy and minimal multi-cell encapsulation.

**Figure S4. Comparison of percentage of cell recovery using different loading methods for microspore encapsulation.** Three strategies were evaluated: (1) tubing-based preloading with the tubing laid flat prior to encapsulation; (2) direct syringe loading with magnetic stirring; and (3) direct syringe loading without mixing. Percentage of cell recovery was quantified as the ratio of the experimental to the theoretical average cell occupancy ($\lambda_{\mathrm{ex}}/\lambda_{\mathrm{th}}\times100$). Tubing-based preloading achieved the highest cell recovery, with λ_ex_ reaching approximately 60% of λ_th_, and was therefore selected for subsequent experiments. Direct syringe loading with magnetic stirring resulted in cell sedimentation within the syringe and non-uniform droplet generation at higher stirring speeds due to movement of the stirring bar, whereas syringe loading without mixing exhibited pronounced cell sedimentation and poor recovery. Bar plots represent mean ± SD from independent experiments.

**Figure S5. Validation of the tubing-based loading strategy for large plant cells.** **(a)** Temporal stability of cell concentration at the chip inlet during continuous droplet generation using the tubing-based loading strategy. Cell concentration remained stable throughout 40 min of operation, with no significant change over time, demonstrating that the tubing-based loading strategy maintained a near-constant input cell concentration during prolonged loading (one-way ANOVA, p > 0.05, n = 4). **(b)** Cell concentration before and after passage through the Tygon loading tubing. No significant difference in cell concentration was observed between the bulk suspension and the loaded cell suspension (p > 0.05, n = 4), indicating negligible cell adhesion to the tubing wall. Error bars represent mean ± SD.

**Figure S6**. **Optimization of FDA dye incubation time for fluorescence viability imaging of cells in droplets.** **(a)** Representative fluorescence images of droplets and encapsulated cells at i. 15, ii. 40, iii. 70, iv. 100, and v. 130 minutes after adding FDA to the oil phase. FDA dye yields fluorescence via both intracellular esterase activity (within live cells) and extracellular diffusion. Prolonged incubation favors extracellular accumulation, decreasing the accuracy of viability discrimination—especially in droplets containing multiple cells, where dead cells may be misclassified as live due to excess background fluorescence. To minimize such errors, incubation times shorter than 130 min are optimal. Consistent timing is critical for reproducibility. **(b)** Signal-to-noise (S/N) ratio versus incubation time, measured from a population of 10 cells (both live and dead) on day 0. S/N ratio peaks at approximately 60 min, providing maximal contrast between cell fluorescence and background. Error bars reflect the variability in signal among live and dead cells. Based on these results, an incubation time of 70 min was selected to achieve robust and reliable cell viability imaging.

**Figure S7.** **Workflow for cell segmentation and fluorescence quantification using ImageJ. (a)** Bright-field image of droplets containing individual cells, used for mask generation. **(b)** Segmentation mask created in ImageJ, delineating cells for subsequent analysis; each colored region corresponds to a detected particle (cell). **(c)** The segmentation mask is applied to the paired fluorescence image to extract quantitative cell data. The procedure involves: (1) applying bright-field commands in ImageJ to find edges, apply Gaussian blur, threshold for focused cells, and analyze particles; (2) generating a mask with cell locations and sizes; (3) applying the mask to the fluorescence image, ensuring consistent gain settings, and extracting area and mean intensity for each cell; (4) quantifying mean intensity, which serves as an indicator of cell viability.

**Figure S8. Effect of reservoir wall geometry on droplet arrangement for imaging (side views).** **(a)** Side view of a straight-edged PDMS monolayer droplet reservoir, where droplets stack against the wall, creating multiple layers and hindering imaging. **(b)** Side view of a curved-edge PDMS reservoir, which promotes formation of a single layer of droplets along the glass surface for high-quality, planar imaging. **(c)** Side view schematic of the 3D-printed plastic mold used to cast the PDMS reservoir, showing the dimensions and curved edge designed to produce the optimal droplet arrangement for imaging.

**Figure S9.** **Carrier oil does not impact cell viability in bulk culture.** Cell viability was assessed by fluorescence microscopy, comparing standard bulk conditions (black bars) with bulk samples containing 50% carrier oil added on top (gray bars). No significant difference was observed between the two conditions across all time points (p > 0.05). Fluorescence microscopy was used as the gold standard for viability measurement. Error bars indicate the standard deviation from independent replicates. More than 80 cells were used for each experiment.

**Figure S10. Closed droplet-fixation platform for longitudinal imaging of plant cells. (a)** Top view of the closed Petri-dish setup containing a PDMS monolayer droplet reservoir, an internal water source for humidity control, and magnetic spacers to prevent condensation on the lid. **(b)** Side view schematic illustrating the evaporation mechanism: i. initial rapid evaporation under an unsaturated vapor environment (Day 0), and ii. progressive vapor saturation leading to a substantially reduced evaporation rate (Days 2–4). **(c)** Experimental measurement and exponential fit of cumulative oil evaporation in the closed Petri dish. **(d)** Validation of the droplet-fixation platform using non-embryogenic B73 × Mo17 cells. i. Comparison of cell viability based on cell size between conventional bulk culture and fixed droplets over four days after fixation, showing no significant differences between the two conditions. ii–iv. Representative bright-field images of the same fixed droplets at Days 0, 2, and 4, respectively. Green and red circles highlight representative live and dead cells. **(e)** Representative embryogenic DAS 139/39-DH cells following transfer from the scalable 3D droplet incubation platform to the fixation system after 14 days of culture. The same embryogenic structures were tracked at i. Day 14+2 and ii. Day 14+4, with no additional embryogenic structures observed, confirming that embryogenesis rate did not increase beyond the standard 14-day endpoint.

Our closed system controls evaporation dynamics, facilitates droplet fixation, and enables longitudinal cell studies within immobilized droplets. In this setup, evaporation initially occurs rapidly due to low vapor pressure, but as water vapor saturates the internal atmosphere, the evaporation rate declines to nearly zero, supporting multi-day cell viability. For droplet fixation, we dispensed B73 × Mo17 cells in droplets overlaid with 500 μL of oil, resulting in droplets that first float on the reservoir surface in circular form. As oil gradually evaporates, droplets settle and become fixed in place, acquiring hexagonal shapes. This immobilization enables precise re-imaging of the same cells over time—a critical advantage for tracking developmental trajectories. However, when oil and medium loss extends beyond six days, cell viability is compromised. We quantified these evaporation dynamics and determined the rate follows an exponential decline, with system parameters derived from experimental fits. To quantitatively validate the fixation platform, cell viability in fixed droplets was compared with conventional bulk culture over four days after droplet fixation. No significant differences in viability were observed between the two conditions, demonstrating that the fixation process itself does not compromise cell viability during longitudinal imaging (Figure S9d(i)). Representative bright-field images further illustrate that some cells gradually shrink while others remain viable, reflecting normal biological heterogeneity rather than an artifact of the fixation method (Figure S9d(ii–iv)). Demonstrating the platform's utility for longitudinal studies, DAS 139/39-DH maize cells cultured for 14 days in 20 nL droplets were transferred to the closed reservoir with 1000 μL oil; by two days, droplets were nearly fixed, and cells remained viable through Day 4, supporting ongoing in-line developmental analysis. No additional embryogenic structures were observed during this extended tracking period (Days 14+2 to 14+4), confirming that embryogenesis did not increase beyond the standard 14-day endpoint (Figure S10e). Overall, this closed Petri dish fixation system enables consistent, multi-day cell imaging and tracking, addressing the core challenges of droplet-based culture systems.

**Figure S11**. **Uniform 200 nL droplets generated with varying cell loading densities.** **(a)** Droplets with average cell loading λ_ex_ = 1.3; **(b)** droplets with λ_ex_ = 2; **(c)** droplets with λ_ex_ = 7. Increasing loading density results in more droplets containing multiple cells. Scale bar: 500 μm.

**Figure S12. Stability of 200 nL droplets during long-term stacked incubation. (a)** Coefficient of variation (CV) of droplet diameter at Day 0 and Day 14, CV values were calculated from 50 droplets per condition; showing no significant change during incubation (p = 0.66, n = 4). **(b,c)** Representative bright-field images of 200 nL droplets at Day 0 and Day 14, respectively, demonstrating that droplets remained discrete and maintained their integrity without observable coalescence during stacked incubation in the cylindrical reservoir. Scale bar: 1 mm.

**Figure S13. Comparison of cell viability in bulk culture, 20 nL droplets, and 200 nL droplets.** **(a)** Viability of non-embryogenic B73 × Mo17 cells during 6 days of incubation in bulk culture, 20 nL droplets, and 200 nL droplets (n = 4). **(b)** Viability of embryogenic DAS 139/39-DH cells during 14 days of incubation under the same culture conditions (n = 4). An inlet cell concentration of 50,000 cells/mL was used for all conditions. Cell viability was assessed using fluorescein diacetate (FDA) staining. No significant differences in cell viability were observed between the 20 nL and 200 nL droplet formats at any evaluated time point (p > 0.05), while viability in both droplet formats was comparable to or higher than that observed in conventional bulk culture. For this experiment, a 10-fold higher FDA concentration than the standard protocol described in the Methods was used to ensure uniform staining of cells encapsulated in 200 nL droplets. Error bars represent SD.

**Figure S14. Stability of non-embryogenic and embryogenic 200 nL droplets after 14 days. (a) Percentage of stable non-embryogenic and embryogenic droplets after 14 days of incubation in the cylindrical reservoir. Droplets with diameters exceeding three standard deviations above the mean diameter of the non-embryogenic droplet population, corresponding approximately to twice the droplet volume, were classified as merged. Approximately 98% of non-embryogenic droplets and 85% of droplets containing embryogenic structures remained stable, with no significant difference between the two groups (*p* = 0.16, *n* = 3 independent experiments). Across the three experiments, a total of 200 non-embryogenic droplets were randomly sampled to establish the stability threshold, and a total of 33 embryogenic droplets were analyzed. Error bars represent SD.**  **(b) Representative bright-field image of a stable 200 nL droplet containing an expanded embryogenic structure at Day 14, with an enlarged view of the indicated region. (c) Representative bright-field image of a merged droplet containing an embryogenic structure at Day 14, with an enlarged view of the indicated region. Scale bars: 1000 μm (overview) and 500 μm (enlarged view).**

**Figure S15. Effect of cell loading density on the viability of encapsulated plant cells. (a) Viability of non-embryogenic B73 × Mo17 cells during 6 days of incubation in 200 nL droplets at different cell loading densities and in conventional bulk culture (n = 4). (b) Viability of embryogenic DAS 139/39-DH cells during 14 days of incubation under the same culture conditions (n = 4). Inlet cell concentrations of 5,000, 10,000, and 50,000 cells/mL were used for the droplet conditions, corresponding to λ_th_ = 1, 2, and 10, respectively, with λ_ex_ reaching approximately 50–60% of λ_th_. Bulk cultures were prepared at a cell concentration of 50,000 cells/mL. Cell viability was assessed using fluorescein diacetate (FDA) staining. Higher cell occupancies maintained viability comparable to conventional bulk culture, whereas lower occupancies showed a trend toward reduced viability over time. For this experiment, a 10-fold higher FDA concentration than the standard protocol described in the Methods was used to ensure uniform staining of cells encapsulated in 200 nL droplets. Error bars represent SD.**

**Figure S16. Determination of the minimum cell number required for accurate viability analysis in bulk culture.** Viability percentage was quantified at **(a)** Day 0, **(b)** Day 2, **(c)** Day 4, and **(d)** Day 6 as a function of the number of analyzed cells. Sampling 80 cells yields a variation of less than 2% compared to analyzing 160 cells across all time points, indicating that 80 cells is sufficient for reliable viability assessment in these experiments. Fluorescence microscopy was used as the gold standard for viability measurement.

**Supplementary Video 1**: 20 nL droplet generation.

**Supplementary Video 2:** 200 nL droplet generation.
